# An evaluation of false-positive rifampicin resistance on the Xpert MTB/RIF

**DOI:** 10.1590/0074-02760170051

**Published:** 2017-11

**Authors:** Yeliz Tanriverdi Cayci, Kemal Bilgin, Ahmet Yilmaz Coban, Asuman Birinci, Belma Durupınar

**Affiliations:** 1 Ondokuz Mayis University, Medical Faculty, Department of Medical Microbiology, Samsun, Turkey; 2 Ondokuz Mayis University, Vocational School of Health Services, Department of Medical Services and Techniques, Samsun, Turkey

**Keywords:** Mycobacterium tuberculosis, rifampicin resistance, Xpert MTB/RIF assay

## Abstract

**BACKGROUND:**

*Mycobacterium tuberculosis* (MTB) is one of the most significant causes of mortality and morbidity. Early diagnose is important especially in multiple drug resistant tuberculosis to avoid transmission. Traditional techniques requires at least one to three weeks for diagnosis of tuberculosis. Diagnostic delays with multiple drug resistant tuberculosis are associated with worse clinical outcomes and increased transmission The Xpert MTB/RIF assay is one of the new diagnostic device for the diagnosis of tuberculosis and rapid detection of rifampicin resistance.

**OBJECTIVE:**

We assessed the performance of Xpert MTB/RIF assay for detecting rifampicin resistance using phenotypic drug susceptibility tests as automated BD MGIT 960.

**METHODS:**

Total of 2136 specimens were included in the study. Xpert MTB/RIF testing was performed on samples, using version 4 cartridges, according to the manufacturer's recommendations. The MTBC culture and first-line phenotypic DST were performed in automated BD MGIT 960 (Becton & Dickinson, USA) according to the recommendations of the manufacturer. Agar proportion was used in the case of inconsistency for rifampicin resistance.

**FINDINGS:**

Thirty-four samples (19 respiratory and 15 nonrespiratory samples) were determined as positive for *M. tuberculosis* complex by Xpert MTB/RIF (Cepheid GeneXpert® System, USA). Xpert MTB/RIF assay detected 4/34 (11.7%) specimens as rifampicin resistant. One of the rifampicin resistant isolates was determined susceptible in MGIT 960 automated system. This isolate was also tested with agar proportion method and found susceptible to rifampicin.

**MAIN CONCLUSION:**

The Xpert MTB/RIF assay can be used as first-line assay for the detection of *M. tuberculosis.* However, microbiologists must be aware of the limitations of the assay.

Tuberculosis (TB) constitutes a serious threat to public health in the world, with nearly 10 million new cases and 1.7 million deaths annually (Chang et al. 2012). The global control of TB and multiple drug resistant TB (MDR-TB) has created an urgent need for timely and effectively diagnostic method (Chang et al. 2012). MDR-TB, defined as TB caused by mycobacteria that are resistant to at least isoniazid and rifampicin (RIF), is associated with worse clinical outcomes, and its treatment is expensive, lengthy, and complex (Zetola et al. 2014). Diagnostic delays with MDR-TB are associated with worse clinical outcomes and increased transmission (Falzon et al. 2011). Traditionally, a diagnosis of MDR-TB infection requires mycobacterial culture and phenotypic drug susceptibility testing (DST) (Zumla et al. 2012). These techniques require relatively advanced laboratory capacity, are labor-intensive, and take 1 to 3 months before the results are available (Zetola et al. 2014). In 2011, the World Health Organization recommended the use of rapid molecular genotyping methods for DST at the initial diagnosis of TB (Falzon et al. 2011). RIF inhibits bacterial DNA-dependent RNA polymerase; encoded by the RNA polymerase gene (*rpoB*). Resistance to this drug has mainly been associated with mutations in a limited region of the *rpoB* gene (Telenti et al. 1993). RIF resistance may occur alone or in association with resistance to isoniazid and other drugs (Steingart et al. 2014). A molecular diagnostics based on detection of mutations in an 81-base pair region of the *rpoB* gene have led to the development of rapid molecular assays for RIF resistance, which is frequently used as a marker for MDR-TB (Telenti et al. 1993). One of these molecular techniques is Xpert MTB/RIF assay (Cepheid, Sunnyvale, CA, USA) which is a rapid, automated, and cartridge-based genotypic test that can simultaneously detect *Mycobacterium tuberculosis* complex (MTBC) and RIF resistance (Helb et al. 2010). Compared to conventional diagnosis methods, the Xpert MTB/RIF assay could detect TB and rifampicin-resistance in one sputum sample within 2 h (Boehme et al. 2010).

In this study, we aimed to evaluate the MTB/RIF assay, with particular attention given to the specificity of the assay for detecting RIF resistance.

## MATERIALS AND METHODS

### Clinical samples

In total, 2136 specimens were included in the study. They originated from patients with suspected TB and were sent for routine mycobacteriology diagnostics between October 2011 and September 2016 at Ondokuz Mayıs University Medical Faculty Mycobacteriology Laboratory. A total of 34 samples (19 respiratory and 15 nonrespiratory samples) that were determined as positive for *M. tuberculosis* complex by Xpert MTB/RIF (Cepheid GeneXpert® System, USA) were included in the study.

### Procedures for Xpert testing

Xpert MTB/RIF testing was performed on samples, using version 4 cartridges, according to the manufacturer's recommendations. The Xpert assay sample reagent (containing NaOH and isopropanol) was added in a 1:3 ratio to the tubes to kill the mycobacteria and liquefy the sample. The mixture was vigorously shaken and allowed to sit for 15 min before being shaken again and allowed to sit for another 5 min. Finally, 2 mL was pipetted into the Xpert assay cartridge and inserted into the GeneXpert instrument for polymerase chain reaction (PCR) testing. The measurement and analysis were conducted automatically and reported by the GeneXpert Dx software (version 4.0).

### Cultures and phenotypic DST

The MTBC culture and first-line phenotypic DST were performed in automated BD MGIT 960 (Becton & Dickinson, USA) according to the recommendations of the manufacturer.

Specimens were decontaminated using sodium hydroxide (NaOH) except sterile body fluids like cerebrospinal fluid. After concentration by centrifugation at 3000 *g* for 15 min, the sediment was resuspended in 1.5 mL of 0.5 M phosphate buffer (pH 6.8) and inoculated in MGIT-7H9 broth supplemented with oleic acid-albumin-dextrose-catalase (OADC) and PANTA (Becton Dickinson). This was incubated using MGIT 960 instrument (Becton-Dickinson and Company, Sparks MD, USA) as well as Lowenstein-Jensen (LJ) medium at 37°C. MTBC strains grown on MGIT medium were tested for drug susceptibility in MGIT 960.

Agar proportion method on 7H11 agar medium was used for the testing of the isolate which was determined resistant to rifampicin in Xpert MTB/RIF and susceptible in MGIT 960, according to standard procedures. The drug concentration used was rifampicin 1.0 μg/mL. The proportion of resistant organisms in the inoculum was calculated by comparing the number of colonies growing on the drug-free medium (minimum number required = 50) with the number growing on drug-containing medium. If > 1% of the inoculum was found to grow in the presence of the critical concentration used, the isolate/strain was regarded as drug resistant.

## RESULTS

The Xpert MTB/RIF assay was detected 4/34 (11.7%) samples as RIF resistant. The resistance was conferred by two different *rpoB* gene mutations in the 81 bp-RRDR of MTB. These were detected by probes D and E. The probe frequency associated with the observed RIF-resistance were as follows: E (3/4), D (1/4) and no RIF-resistance was associated with probe A, B and C.

However, in MGIT 960 automated system, RIF resistance was detected in 3/34 isolates (Table I). The isolate that determined as resistant to RIF in Xpert MTB/RIF but susceptible in MGIT 960 automated system, was a sputum sample and also tested with agar proportion method for RIF resistance and found susceptible to RIF. Also that isolate was determined resistant to streptomycin, isoniazid and susceptible to ethambutol by MGIT 960. The other three isolates which were resistant to RIF both in Xpert MTB/RIF and in MGIT 960 found resistant to streptomycin, isoniazid and ethambutol by MGIT 960 system (Table II).

**TABLE I:** Results of rifampicin (RIF) resistance in Xpert MTB/RIF assay and MGIT 960 system

		MGIT 960	
		Resistant	Susceptible
Xpert MTB/RIF	Resistant	3	1
	Susceptible	0	30

**TABLE II:** Specimens with rifampicin (RIF) resistance as detected by the Xpert MTB/RIF assay

Specimen	Type	Basis of rifampicin resistance as detected by the MTB/RIF assay	Rifampicin phenotypic susceptibility result (1.0 mg/L)
1	Bronchoalveolar lavage	Prob E did not bind	R
2	Exudate	Prob E did not bind	R
3	Gastric fluid	Prob E did not bind	R
4	Sputum	Prob D did not bind	S

## DISCUSSION

The need for rapid and reliable methods for the diagnosis of tuberculosis has led to molecular diagnostic methods becoming widespread and taking a strong and complementary role along with conventional tests (Ozyurt 2012). By Xpert MTB/RIF assay, *M. tuberculosis* complex and rifampicin resistance can be determined in a single test in a short time (less than 2 h) directly from patient material via a semiquantitative nested real-time PCR method (Durmaz 2010).

The most common RRDR *rpoB* gene mutations in the 81 bp were in codons 531 (58%), 513 (25%), 526 (8%), 511 (8%), and none for codon 522. These were designated by probes E, B, D, A, and C respectively on the Xpert MTB/RIF assay (Mboowa et al. 2014). A study by Yue et al. (2003) found these frequencies 531 (41%), 526 (40%), and 513 (4%) in China. In a review by Steingart et al. (2014) the pooled sensitivity and specificity by univariate analysis were 95% [95% credible interval (CrI) 90% to 97%] and 98% (95% CrI 97% to 99%), respectively for RIF resistance detection at Xpert MTB/RIF.

Lately, molecular-based studies have reported that TB may be caused by multiple strains in the same patient. Far from being uncommon, mixed MTBC infections have been reported in up to 50% of TB cases from certain settings in which TB is endemic (Van Rie et al. 1999, 2005, Glynn et al. 2004, Cohen et al. 2011, 2012, Huyen et al. 2012). While the Xpert MTB/RIF assay has the ability to simultaneously test for a larger number of *rpoB* mutations, it is not able to detect all mutations that cause RIF resistance (Blakemore et al. 2010, Helb et al. 2010). Prior studies reported that some isolates with positive Xpert MTB/RIF but negative phenotypic DST results for RIF resistance (generally considered Xpert MTB/RIF false positives) in fact have mutations associated with a RIF-resistant phenotype (in spite of repeated and consistent negative results for RIF resistance on phenotypic) suggesting that some supposedly false-positive Xpert MTB/RIF test results may be accurate and actually reflect insufficient phenotypic DST sensitivity (Boehme et al. 2011, Van Rie et al. 2012).

Zetola et al. (2014) has showed in their study that the presence of wild-type sequences, which can be detected by the probes, with resistant sequences may make this situation intrinscally more susceptible to false-negative results in the mixed target MTBC populations.

Blakemore et al. (2010) evaluated the analytical performance of Xpert MTB/RIF assay in their study and tested the ability of the assay to detect the RIF-resistant fraction of a mixed sample *(M. tuberculosis* DNA with a wild-type *rpoB* sequence was mixed in various ratios with *M. tuberculosis* DNA that contained *rpoB* mutations). Their study showed that the proportion of mutant DNA required for the detection of rifampicin resistance was dependent on the type of mutation. Xpert MTB/RIF assay is capable of detecting the presence of RIF resistance mutations down to a concentration of 40% mutant DNA. Chakravorty et al. (2012) demonstrated that detection of RIF resistance in DNA mixtures with 10, 20, and 30% mutant DNA were indistinguishable from a sample containing 100% wild-type DNA.

Theron et al. (2011) reported six samples as RIF resistant Xpert MTB/RIF and MGIT 960 identified five of these isolates as susceptible to RIF. Five out of six samples were found resistant by sequencing and/or MTBDRplus. The studies showed that the difficulty of distinguishing false-positive from true-positive RIF-resistant results, particularly in clinical practice (Van Rie et al. 2012).

In conclusion, the Xpert MTB/RIF assay has become a valuable first-line assay for the detection of *M. tuberculosis*. However, microbiologists and clinicians must be aware of the limitations of the assay when interpreting the Xpert MTB/RIF test results because mixed MTBC infections may be responsible for false-positive and -negative results. Further studies are needed to evaluate the performance of the Xpert MTB/RIF assay for the detection of RIF resistance in a range of clinical settings and large number of specimens.
